# Current Progress in the Chemoenzymatic Synthesis of Natural Products

**DOI:** 10.3390/molecules27196373

**Published:** 2022-09-27

**Authors:** Evan P. Vanable, Laurel G. Habgood, James D. Patrone

**Affiliations:** 1 Department of Chemistry and Biochemistry, Elmhurst University, Elmhurst, IL 60126, USA; 2 Department of Chemistry, Rollins College, Winter Park, FL 32789, USA

**Keywords:** chemoenzymatic, natural product synthesis, biocatalysis

## Abstract

Natural products, with their array of structural complexity, diversity, and biological activity, have inspired generations of chemists and driven the advancement of techniques in their total syntheses. The field of natural product synthesis continuously evolves through the development of methodologies to improve stereoselectivity, yield, scalability, substrate scope, late-stage functionalization, and/or enable novel reactions. One of the more interesting and unique techniques to emerge in the last thirty years is the use of chemoenzymatic reactions in the synthesis of natural products. This review highlights some of the recent examples and progress in the chemoenzymatic synthesis of natural products from 2019–2022.

## 1. Introduction

The biodiversity of organisms from plants to microbes to mammals on Earth has led to a vast wealth of natural products. Throughout history from ancient civilizations to our contemporary one, these natural products have been an invaluable source of bioactive molecules capable of improving their quality of life. Natural products and their derivatives found success in modern drug discovery for a wide range of disease states ranging from diabetes and cardiovascular disease to viral infections and inflammatory diseases with notably high success as antibiotic and anticancer agents [1]. Despite the continued success of natural products in the clinical setting, the pharmaceutical industry divested resources from their discovery in the 1990s due to challenges associated with the rediscovery of known chemical entities, target deconvolution, and resources being allocated to alternative methods of drug discovery [2,3]. More recently there has been a resurgence in natural product discovery, structure elucidation, and progression of natural products to the clinic as a consequence of increased resources and advances in methodologies.

The field of natural product synthesis dates back to 1828, fascinating and inspiring generations of chemists [2,4]. Natural products are often characterized for their high structural complexity stemming from an enriched number of stereocenters, sp^3^ carbons, oxygen atoms, and rigid carbon skeletons as compared to synthetically designed molecules [1]. The combination of the rich, diverse, and structurally complex structures of natural products and the drive, creativity, and talent within the synthetic community makes the synthesis of natural products one of if not the most important fields for both training chemists and developing novel synthetic methods [4]. The pursuit of these diverse targets has seen the field of organic chemistry expand its capabilities in leaps and bounds in areas such as but not limited to retrosynthetic analysis, stereoselective and regiospecific C-C bond formations, cascade reactions, orthogonal protecting groups, protecting group free synthesis, organometallic catalysis, convergent synthesis, atom efficiency, and green chemistry [4]. To this point, many modern organic techniques have been applied to natural product synthesis. For example, organometallic mediated C-H activation bond activation chemistry (directed and non-directed) such as in the synthesis of (−)-epicoccin G and artemisinin [5]. The boundaries and application of electrochemical reactions such as decarboxylative couplings have been extended into to the synthesis of *R*-(Z)- nerolidol [6]. Photochemical reactions such as cycloadditions, arene couplings, and C-N bond formations are an emerging methodology in the synthesis of natural products such as (−)-pavidolide B, (+)-flavisiamine F, and (+)-iosocorynantheol [7].

Over the past twenty years, chemists have been going back to nature and its biosynthetic pathways to develop new advantageous bond forming methodologies through chemoenzymatic syntheses [8,9,10,11,12,13,14]. These pioneering scientists have enriched our synthetic landscape across numerous reaction types such as chiral resolutions (many of the first applications of chemoenzymatic processes), saponifications, hemiacetal formations, oxidations and reductions, and C–C bond forming reactions as well as classes of molecules including glycans, peptides or derivatized amino acids, polyketides, and terpenoids. The benign nature, stereospecificity, and potential of chemoenzymatic processes has led researchers to invest heavily in their development.

As chemoenzymatic methods became more widely available and applicable, their benefits to the synthetic communities are greater than just expanded methodologies. Enzyme-catalyzed reactions incorporate the majority of the twelve principles of green chemistry that seek to reduce our impact on human health and the environment [15]. Enzymes are inherently non-toxic and natural (less hazardous chemical synthesis and use of renewable feedstocks). Their catalytic nature affords reactions that can be run at ambient to slightly elevated temperatures in biphasic or completely aqueous media (catalysis, design for energy efficiency, safer solvents and auxiliaries) and impart regio- and stereoselectivity (atom economy, waste prevention) [16]. Since chemoenzymatic methods combine high regioselectivity and stereoselectivity with environmental and cost benefits, they are attractive method for large scale synthesis and as such have been adopted for the synthesis of several high value pharmaceutical agents such as sitagliptin, simvastatin, and darunavir [16,17].

The continued application and success of chemoenzymatic syntheses in these settings has continued to fuel the diversity and pace of research into biocatalytic approaches. This research has produced advances in the variety and number of chemoenzymatic processes and increased their capabilities through scalability, multiple enzyme cascades, and flow processes. The importance of the chemoenzymatic synthesis of natural products can be seen in the explosion of recent syntheses and review articles highlighting their accomplishments [11,18,19,20,21,22,23,24,25,26,27,28,29,30]. This report is organized by classification of molecule and aims to highlight the diversity and power of this field through selected chemoenzymatic syntheses of natural products from 2019–2022.

## 2. Selected Natural Product Syntheses Incorporating Chemoenzymatic Methods

### 2.1. Terpenoids

**Scheme 1:**
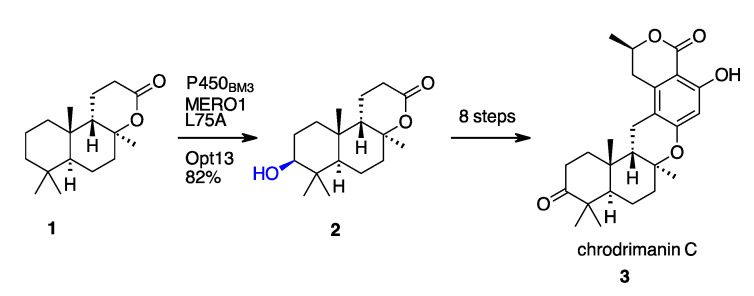
Selective chemoenzymatic hydroxylation towards the synthesis of chrodrimanin C (**3**) [32].

One of the principal scientists featured throughout this review, Hans Renata, pushes the boundaries of the utility and elegance of chemoenzymatic synthesis across multiple complex classes of molecules. The work of the Renata group is often impressive in its nuanced design which is integrated within traditional synthetic sequences [20,22,23,31,32,33]. In a recent paper they disclosed the synthesis of chrodrimanin C (**3**), verruculide A, and polysin using multiple chemoenzymatic steps (**Scheme 1**) [33]. A key step featured in these syntheses is an enzymatic hydroxylation of a 6,6,5 or 6,6,6, steroid core, intermediate 1 in the case of chrodrimanin C (**3**). These reactions were performed on gram scale, 67 & 83% yields, depending on starting material, selectivity for oxidation of a single methylene despite the presence of 6 or 7 other oxidizable methylene groups, and with enantioselectivity of course. This scale is an impressive feature for chemoenzymatic methods, considering the importance of this feature for transformations in total synthesis.

**Scheme 2:**
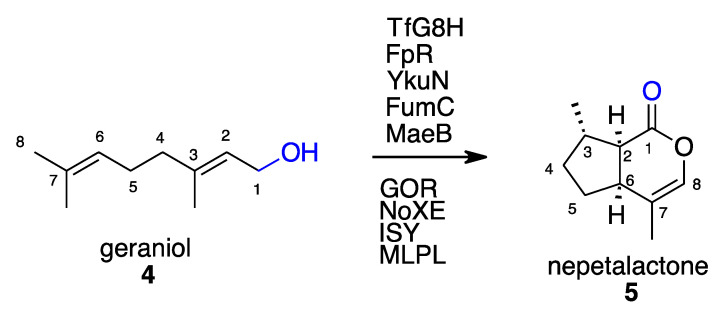
One-pot multienzyme cascade synthesis of nepetalactone (**5**) from geraniol (**4**) [34].

Tang and co-workers’ synthesis of the bicyclic terpenoid nepetalactolone, the active molecule in catnip and a natural insect repellent, features a one-pot multienzyme (OPME) system that is stereoselective setting three contiguous stereocenters while utilizing geraniol (**4**) as a precursor (**Scheme 2**). [34]. This synthesis features a ten-enzyme cascade, half of which are necessary to perform the requisite biosynthetic steps, and half of which are required for auxiliary needs or cofactor regeneration. The chemical steps performed by the enzymes are allylic hydroxylation, alcohol oxidation, aldehyde reduction, cyclization, and a hemiacetal oxidation. One of the more elegant aspects of this system is the ability to perform oxidative and reductive steps in the same pot, with the same NAD/NADH system. Although the experiments were run on a small scale, the yields are excellent (93%) with potential to produce approximately 1 g nepetalactone per liter of solution at a reasonable cost (<$120/g).

**Scheme 3:**
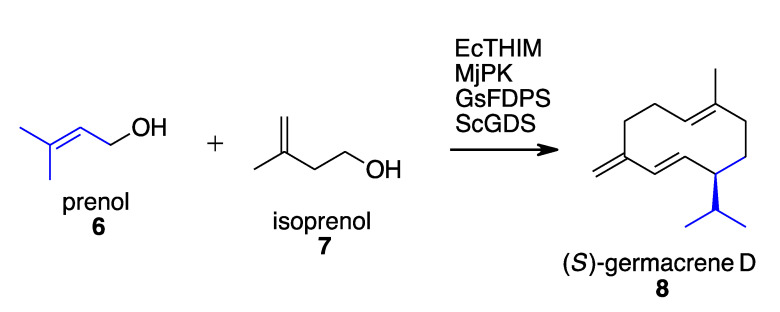
OPME system utilized in the activation, condensation, and cyclization of prenol (**6**) and isoprenol (**7**) for the synthesis of (*S*)-germacrene D **(8**) [35].

A novel method using an OPME cascade of enzymatic reactions to synthesize triterpenes of highly varied structures, including cyclized variants was recently reported by Allemann and coworkers [35]. Noteworthy is that the scope of starting material, enzymatic variance, and enzymatic combinations, as many as four enzymes total, all within a OPME framework to generate simple but highly varied triterpenoids. The enzymatic transformations utilized include monophosphorylation by *Ec*THIM, diphosphorylation by *Mj*IPK, synthesis of natural and unnatural farnesyl diphosphosphates by *Gs*FDPS, and cyclization and/or bicyclization using a variety of enzymes. Pyruvate kinase (PK) acts as a supplementary enzyme to replenish the ATP substrate pool throughout the phosphorylation reactions. Seven sesquiterpenoid compounds, many first reported in this study, and the antibacterial/antifungal (*S*)-germacrene D (**8**) are synthesized. Prenol (**6**) and isoprenol (**7**) were mixed in a 1:2 ratio with *Ec*THIM, *Mj*IPK, PK, *Gs*FDPS, and ScGDS to yield germacrene D (**Scheme 3**). Advantages of their methodology include using less expensive 4- or 5-carbon starting materials and producing both natural and unnatural products in a modular fashion on a milligram scale.

### 2.2. Polyketides

The area of chemoenzymatic synthesis to produce polyketide natural product targets is so critical that it can be said that it is the driver of advancements in the field as a whole. Alison Narayan and David Sherman have been and will continue to build on their pioneering work [14,18,26,28,36,37,38,39,40]. The importance of these two scientists to the field is evidenced by the previous coverage in the literature, including other reviews. Therefore, this work will not include it but allow for interested readers to explore it within these references.

**Scheme 4:**
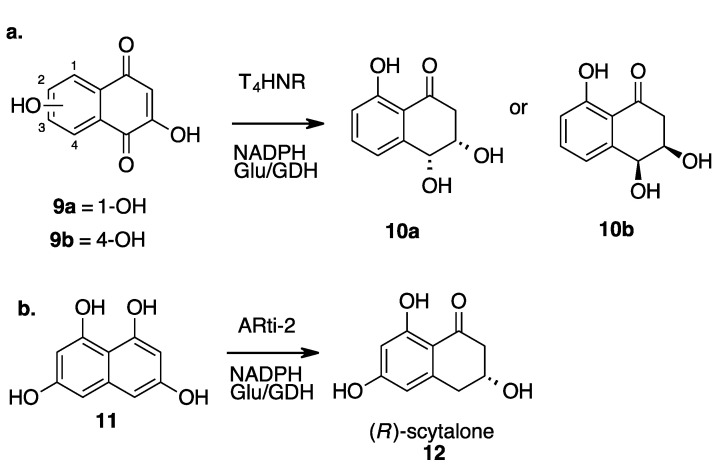
(**a**) Stereoselective chemoenzymatic reductions using T4HNR to form polyketide metabolites **10a**, **10b**. (**b**) Stereoselective chemoenzymatic reductions using Arti-2 to synthesize (*R*)-scytalone (**12**) [41,42].

Stereoselective reductions of simple organic moieties are an easy way to introduce stereocenters: if it can be done. To afford the desired diol products selectively, Husain et al. have applied the use of T_4_HNR to reduce ketones and enols selectively in naphthol systems (**Scheme 4a**) [41]. Intriguingly this process reacts very differently with 2-hydroxy and 3-hydroxyjuglone starting materials. The phenol orients the molecule within the enzyme active site to provide the selectivity for the adjacent ketone to be reduced. While exhibiting a high level of selectivity, the reduction of 3-hydroxyjuglone affords an 82:18 d.r. for **10a** and **10b** which is comparatively modest for an enzymatic transformation. Building off this initial strategy, the Husain group recently reported the small-scale synthesis *(R*)-scytalone (**12**) from simple accessible starting materials using the anthrole reductase ARti-2 and a NADPH cofactor (**Scheme 4b**) [42]. Notable about this chemoenzymatic transformation is that scytalone, generated by the desymmetrization of a perfectly flat tetrahydroxynaphtalene in a stereoselective fashion, also includes another phenol, which is oxidized to a ketone. Despite a small scale and modest yield (23%), the selectivity was >99% for the observed stereoisomer is exceptional.

**Scheme 5:**
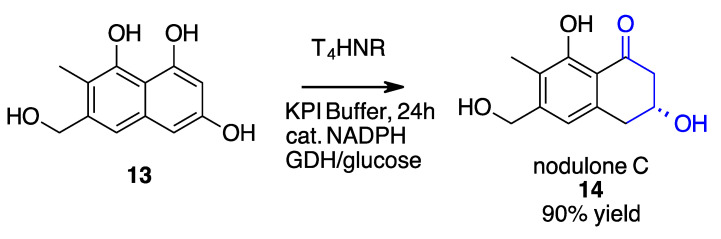
High yielding stereoselective reduction in the synthesis of nodulone C (**14**) [43].

Husain and coworkers continued studies utilizing a system of T_4_HNR, NADPH, and glucose with GDH to synthesize polyketide natural products in the nodulone family (**Scheme 5**) [43]. The synthesis of both nodulone C (**14**) and an unnatural diastereomer of nodulone D are featured. In the case of nodulone D, two stereocenters were set with near perfect d.r. Their ability to doubly hydrogenate the hydroxynapthoquinone selectively, while leaving a benzylic ketone untouched, would be difficult to duplicate using traditional synthetic organic techniques as overreduction would be facile. In nodulone C they once more selectively reduced a hydroxynaphthalene to a phenol, enacting a single enol reduction in a naphthalene with three hydroxy groups selectively in an excellent 90% yield.

**Scheme 6:**
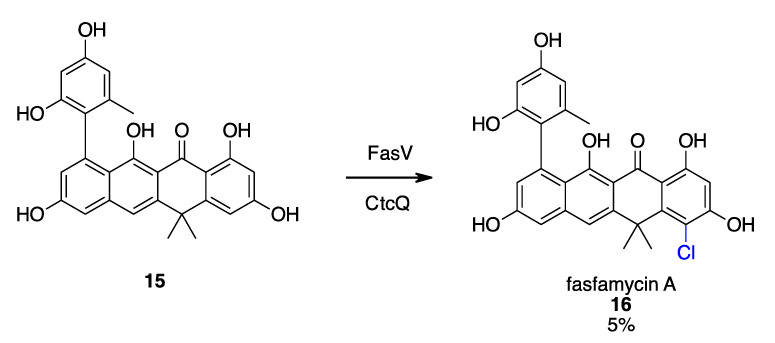
Regioselective halogenation for the synthesis of fasamycin A (**16**) [33].

A recent synthesis of fasamycin A (**6**) from the precursor naphthacemycin B1, utilizing a highly unusual enzymatic halogenation, was recently reported by the Renata group (**Scheme 6**) [33]. The report involved a convergent synthesis that culminated with a halogenation via a chemoenzymatic system that contained a flavin-dependent halogenase, CtcQ as a reductase, Opt13 to regenerate NADH, and NADH/NADPH. The success of the synthesis hinges on a single halogenation of a polyphenol (**15**), at a specific site, with regioselectivity to afford the product in 5% yield. There are 4 rings in precursor (**15**) which could be halogenated, two of which are almost identical electronically and sterically making the regioselectivity achieved even more impressive. The author notes that low yield has been previously reported with halogenases and that enzyme engineering may assist with the issue. Progress in the area of halogenases as a whole will allow this methodology to be used by the broader synthetic community.

### 2.3. Glycans

Glycans are a diverse set of natural products whose size and purpose vary greatly. The range in size from small monosaccharides to enormous polysaccharides possessing hundreds of glycan units correlates with their variety of biological targets and purposes of sugars. Given their versatility, they are used in multiple fields such as food chemistry, medicinal chemistry, and investigations of fundamental biological processes [44,45,46,47,48,49,50,51,52,53].

**Scheme 7:**
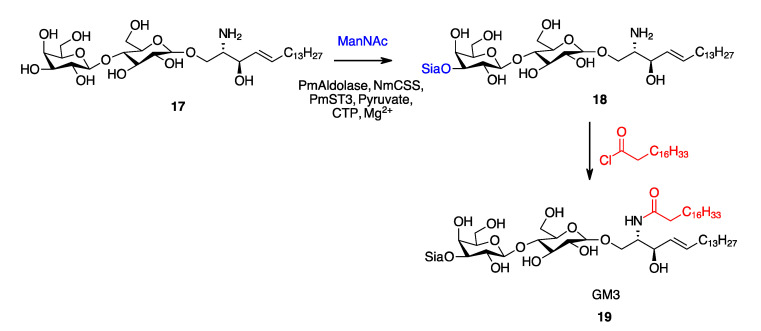
Chemoenzymatic installation of sialic acid in the synthesis of GM3 (**19**) [54].

The Chen group has continued their focused efforts to improve synthetic routes to create structurally diverse libraries of gangliosides, specifically GM3 (**19**) [54]. Comprised of glycan and lipid moieties, GM3 has been implicated as a risk factor in metabolic diseases as well as placed on a prioritized cancer antigen list. An OPME strategy was employed to install sialic acid variants on lactosyl sphingosine (LacbSph) followed by subsequent acylation of a fatty acyl chain to form multiple GM3bSph gangliosides (**Scheme 7**). The six sialic acid variants (ManNAc) were attached to LacbSph forming the GM3 sphingosines in high yields (85–95%) utilizing a OPME approach containing three enzymes, including PmNanA (*P. multocida* sialic acid aldolase), NmCSS (*N. meningitis* CMP-sialic acid synthetase), and PmST3 (*P. multocida* a2-3 sialyltransferase). Subsequent acylation with stearoyl chloride (98–100%) or alternate fatty acyl chains (98–100%) produced ten GM3 gangliosides. Advantages of the synthetic strategy include gram-scale production of LacbSph from an L-serine derivative with minimal purification and efficient mg scale (average 25 mg) production of diverse GM3 gangliosides with fluorine, azide, and diazirine sialic acid derivatives.

**Scheme 8:**
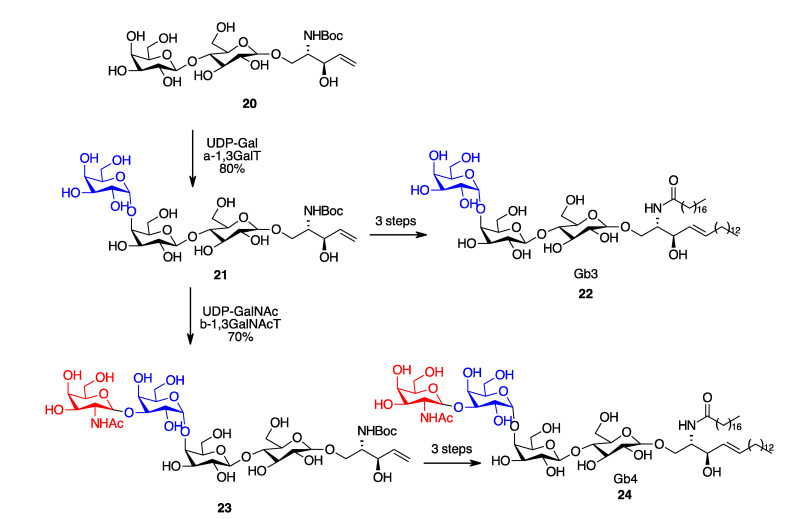
Variable chemoenzymatic glycosylation strategy for the synthesis of Gb3 (**23**) and Gb4 (**24**) [55].

Glycosphingolipids (GSLs) comprised of a glycan and ceramide component are a major component of the cell membrane and are notable signaling molecules essential to numerous biological processes and diseases. Future studies related to mechanisms of these processes, diseases, and applications are contingent on the ready availability of pure and structurally characterized GSLs. To meet this need, the Guo group envisioned a diversity-oriented strategy involving chemoenzymatic glycan synthesis in conjunction with the chemoselective modification of the sphingolipid chain [55]. A series of eight natural and non-natural GSLs were synthesized including Gb3 (**22**), Gb4 (**24**), GM3, and GD3, all of which are known cancer biomarkers. The synthesis of Gb 3 starts with the core intermediate of the strategy being diversified enzymatically by adding Gal using an α-1,4-galactosyltransferase to form the trisaccharide (**21**). The trisaccharide is the chemically modified via a Grubbs-Hoveyda-II catalyzed cross metathesis, Boc removal, and amide formation via an acyl chloride to cleanly yield the fully elaborated GSL Gb3 (**Scheme 8**). The strength of this strategy is its readily amenable to other targets with the same core intermediate and route/steps being utilized with an extra enzymatic step to further diversify the glycan with GalNAc to a tetrasaccharide (**23**) before the chemoselective transformations to yield Gb4 (**24**).

**Scheme 9:**
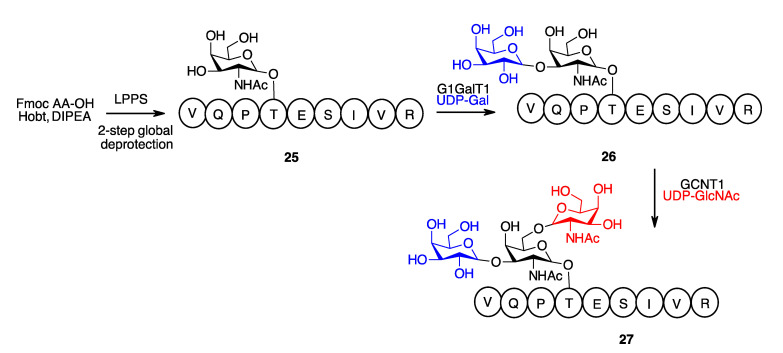
Enzymatic diversification of core peptide for the synthesis of SARS-CoV-2 O-glycopeptides **26** and **27** [56].

Glycopeptides are another class of glycan-based molecules that have implications in normal cellular signaling and disease progression. Again, a major issue with conducting proper studies to understand the biological underpinnings of these molecules is the difficulty of obtain sufficient quantities of pure homogeneous samples. The Li group devised a robust streamlined chemoenzymatic approach to the synthesis of 16 well-defined SARS-CoV-2 O-glycopeptides, 4 complex MUC1 glycopeptides, and a 31-mer glycosylated glucagon-like peptide-1 [56]. Using the SARS-CoV-2 O-glycopeptides as an example, the authors utilized a combination of liquid-phase peptide synthesis (LPPS) and chemoenzymatic glycan synthesis (**Scheme 9**). First the authors used LPPS to build the core 9mer peptide on a 105 mg scale. This was an efficient process using only 1.2 equivalents of amino acid and coupling reagents and leveraging a hydrophobic tag for quick purification by centrifugation and removal of supernatant liquid. Once the 9mer was constructed with the first glycan unit (GalNAc) attached to the T residue a 2-step global deprotection of all sugar, amino acid protecting groups, and the hydrophobic tag yielded the clean core glycosylated peptide. Enzymatic diversification of the GalNAc moiety through the use of varying combinations and orders of glycosyltransferases including C1GalT1, ST6GalNAc1, ST6Gal1, Pd2, 6ST, ST3Gal1, ST3Gal4, GCNT1, B4GalT1 allowed for the formation a and b glycosidic bonds at varying positions with varying substrates to quickly form highly complex glycans highlights the power of this technique.

### 2.4. Peptides and Amino Acids

Peptide and amino acid-based natural products have been some of the most versatile and important natural products used in the clinical setting including molecules such as Vancomycin and Insulin [57]. As such, there is a rich library of literature involving their syntheses and specifically their chemoenzymatic syntheses [58].

**Scheme 10:**
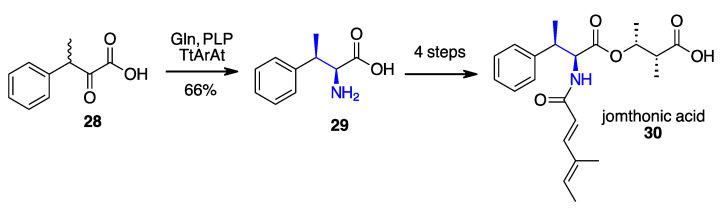
Biocatalyzed DYKAT within the synthesis of jomthonic acid (**30**) [31].

An area which has been developing recently in chemoenzymatic synthesis is the use of enzymes to create stereocenters in small molecules which can be used as a new “chiral pool” to work from towards natural product synthesis. Commonly this is done by dynamic kinetic resolution (DYKAT) or by enzymatic reductions to make enantiomerically enriched alcohols. A recent Renata publication in this area showcases this trend by performing a DYKAT, completed by an enantioselective reductive amination to set two stereocenters: one which was epimerized, one which was generated by the reduction [31]. This reductive amination is actually a transamination from sacrificial glutamine. The scope of this DYKAT was shown through 25 molecules with varying aryl substitutions, one of which was elaborated over four steps to complete the first synthesis of jomthonic acid (**Scheme 10**) (**30**). Significantly, a scaleup to a half gram with >20:1 d.r. was shown by the authors.

**Scheme 11:**
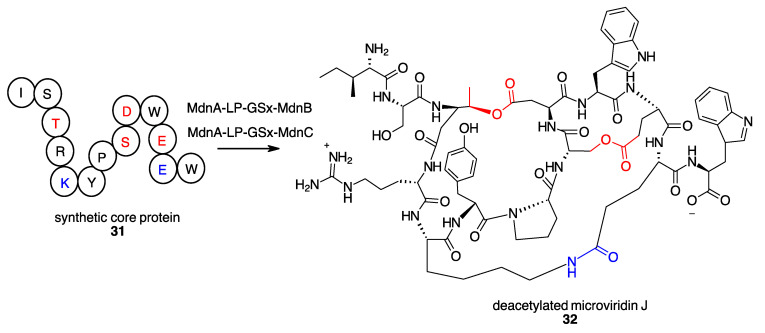
Chemoenzymatic lactonization and lactamization for the synthesis of deacylated microviridin J (**32**) [59].

Bruner and coworkers disclosed a recent strategy to synthesize deacetylated microviridin J (**32**) and explore the activity of engineered enzymes MdnB and MdnC, which perform the tricyclization of the 13mer MdnA core peptide sequence (**Scheme 11**) [59]. Fusion expression constructs were engineered with the MdnA leader peptides crosslinked to both MdnB and MdnC, using varying lengths of glycine/serine linkers (GS_n_, *n* = 5, 10 & 15). This strategy allows for cyclizing just the synthetically produced core 13mer MdnA since the 36 AA leader sequence is already in place on MdnB and C rendering them constitutively active. Upon incubation of these various engineered enzymes with the core peptide, it was found that GS_n_ *n* = 10 & 15 provided the necessary length and flexibility for efficient tricyclization to deacetylated microviridin J. This strategy is an excellent example of engineering and expressing the necessary enzymes for complex macrocyclizations that allowed for a much simpler synthesis of the 13mer core protein versus the endogenously expressed 39 AA leader and core peptide.

**Figure 1:**
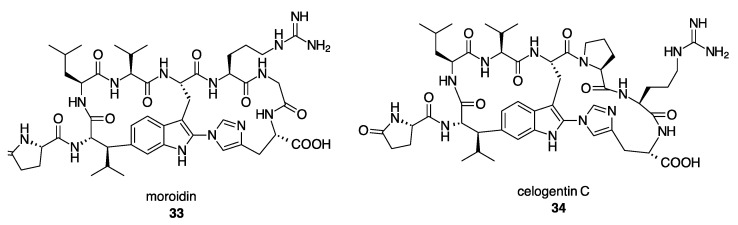
Structures of the peptide-based moroidin (**33**) and celogentin C (**34**) synthesized *in planta* [60].

*In planta* syntheses of moroidin (**33**, previously unsynthesized), and celogentin C (**34**, previously synthesized in 23 steps) were recently reported by the Weng group (**Figure 1**) [60]. Intriguingly, they did this by cloning a gene from *K. Japonica*, the predicted precursor gene for Moroidin, and then expressing it in tobacco. They were able to then grow the tobacco with this newly inserted gene, and modified versions thereof, to produce different extractable natural products on the ~10 mg scale. The only synthetic organic chemistry performed during this synthesis was by the plant itself—enforced by the cloned gene.

### 2.5. Alkaloids

Alkaloid natural products have a rich history as both biologically active molecules and synthetic targets. This class of molecules has also proven to be a remarkable boon for chemoenzymatic syntheses [61,62]. Several syntheses are highlighted here to give exemplars of the diversity of molecule structure and enzymatic reaction. However, as there is not enough space in this report for a thorough coverage of the breadth of the syntheses, an alkaloid specific review can be found in by Cigan et al. [27].

**Scheme 12:**
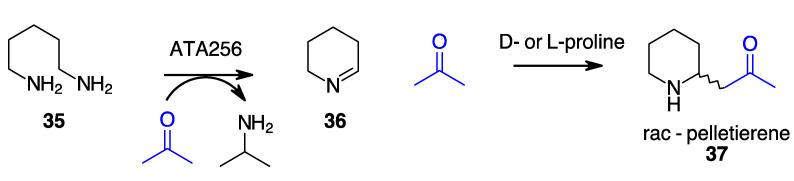
ATA256 biocatalyzed transamination reaction for the synthesis of pelletierene (**37**) [63].

Taday et al. published a hybrid bio-organocatalytic approach to the synthesis of the small piperidine-based natural product pelletierene (**Scheme 12**) (**37**) [63]. This work built upon a previously reported elegant one-pot 2-biocatalytic step approach to norsedaminone that utilized cadaverine, a transaminase, CalB, and a decarboxylative Mannich reaction to synthesize 14 different alkaloids but was unable to synthesize pelletierene [64]. The authors developed a system where transaminase ATA256 generated the reactive imine intermediate (**36**) with acetone playing the dual role as the nitrogen acceptor in this biocatalytic step as well as the nucleophile in the subsequent organocatalyzed Mannich reaction to yield the desired pelletierene. This system was optimized to produce pelletierene in 60% yield with 85 mg isolated. The only weakness of the system is the natural product was isolated as the racemate despite using D- or L-proline in the system. Based upon the lack of difference in ee for the proline isomers, the authors conclude this was most likely due the piperidine racemizing after the reaction [65]. The authors have established a sound system and now are looking to expand the scope of hybrid bio-organocatalytic approaches and further optimize their system to an in vivo model.

**Scheme 13:**
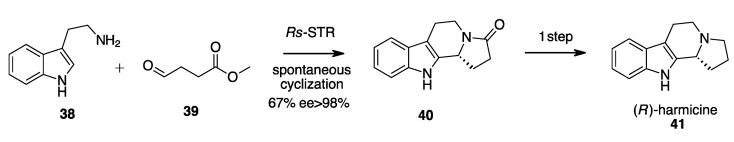
Synthesis of (*R*)-harmicine (**41**) via chemoenzymatic Pictet-Spangler reaction [66].

Indole containing alkaloids are abundant throughout nature and often serve as biologically relevant scaffolds. As such there has been an exciting recent push into the utilization of Pictet-Spangelrases for the synthesis of natural products. The Kroutil group published a concise 2-step chemoenzymatic synthesis of (*R*)-harmicine (**Scheme 13**) (**41**) [66]. The authors were exploring the substrate scope for non-natural substrates for strictosidinesynthases (STRs), an important class of Pictect-Spangelerases that could be leveraged for natural product synthesis. Four STRs from different organisms were cloned and expressed in *E. coli*. The best result was obtained by deleting the signal peptide and adding an N-terminal His-tag. Utilizing the STR from *Rauvolfia serpentina*, tryptamine (**38**) and methyl-4-oxobuta-noate (**39**) were enzymatically condensed with concomitant cyclization to form product (**40**) in 67% yield with >98% ee on 75 mg scale. Smooth reduction of the carbonyl yielded the desired (*R*)-harmicine in a total yield of 62% with >98% ee. This report highlights the power of the enzyme via the concise high yielding synthesis as well the potential for a broad applicability for the future of other targets.

**Scheme 14:**
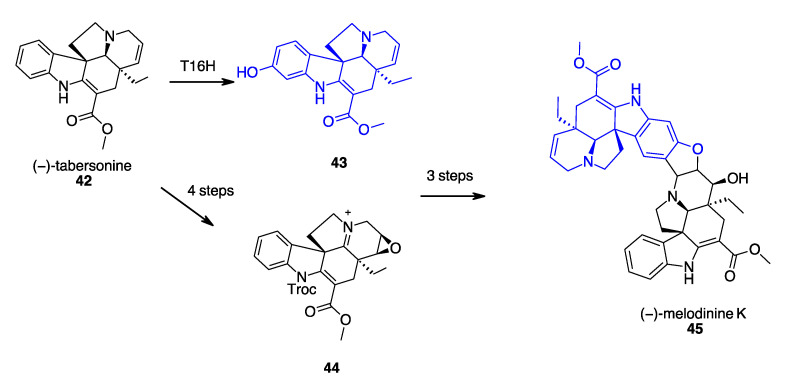
Convergent synthesis of (−)-melodinine K (**45**) featuring chemoenzymatic oxidation of isolated biosynthetic precursor, (−)-tabersonine (**42**) [67].

A 2020 report from the Andrade lab details the first synthesis of the complex bis-indole (−)-melodinine K (**45**) via a convergent chemoenzymatic synthesis (**Scheme 12**) [67]. The authors were cognizant of both the efficiency and sustainability of this synthesis and thoughtfully devised their scheme based on the isolation of 1.6 g complex biosynthetic precursor (−)-tabersonine (**42**) from *V. africana* seeds (**Scheme 14**). Beyond the isolation of the carbon skeleton, a critical biotransformation of (−)-tabersonine (**42**) was employed utilizing the cytochrome P450 monooxygenase tabersonine 16-hydroxylase (T16H) [68,69]. A modified yeast strain, *Saccharomyces cerevisiae* (WAT11 strain) was engineered, and the reaction conditions optimized to allow the site-selective oxidation of (−)-tabersonine (**42**) to (−)-16-hydroxytabersonine (**43**) in 64% yield on the gram scale. (−)-Tabersonine (**42**) is converted to activated epoxide (**44**) in four steps, followed by dimerization with a modified (−)-16-hydroxytabersonine intermediate, which underwent two more synthetic steps to obtain the final product (−)-melodinine K. This synthesis highlights both the power and efficiency of isolating a complex precursor and the selective and efficient site selective chemistry of chemoenzymatic syntheses.

### 2.6. Miscellaneous

As chemoenzymatic synthesis has expanded, there are many interesting natural products and syntheses that fall into molecule classes outside of those listed above that are noteworthy and deserve highlighted in this report.

**Scheme 15:**
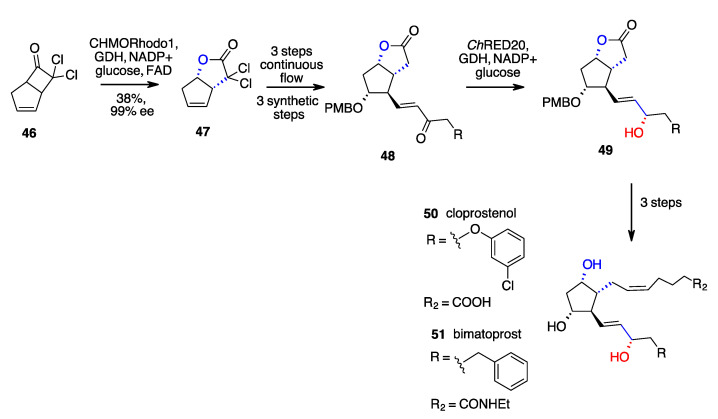
Synthesis of cloroprostenol (**50**) and bimatoprost (**51**) via a combination of synthetic chemistry, flow chemistry, and two stereochemical chemoenzymatic steps [70].

Prostaglandins (PGs) are lipid-based hormone-like signaling molecules that play multiple functions in humans and several such as cloprostenol (**50**) and bimatoprost (**51**) are marketed drugs for veterinary purposes and antiglaucoma treatment, respectively. The Chen lab devised a divergent flow-based chemoenzymatic synthesis capable of producing both cloprostenol and bimatoprost and three other PGs [70]. This synthesis a powerful combination of synthesis, biocatalaysis and flow chemistry that utilizes 11–12 steps from a common starting material to synthesize five high value PGs (**Scheme 15**). The strategy is highlighted chemoenzymatically by a novel stereoselective oxidation to lactone 47 in 99% ee by a Baeyer-Villager monooxygenase (BVMO) and a diastereoselective reduction in 87:13 to 99:1 d.r. by a ketoreductase (KRED) to alcohol 49. From here three synthetic transformations yield the desired prostaglandins. The authors have demonstrated two unique biotransformations that are responsible for setting stereocenters with high ee and d.r., respectively.

**Scheme 16:**
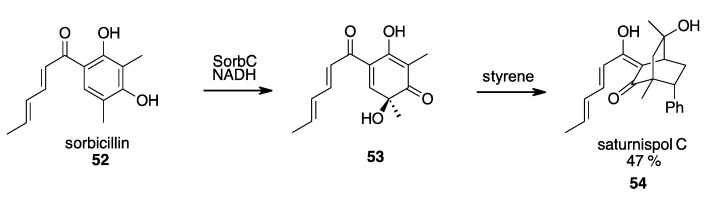
Stereoselective chemoenzymatic oxidation towards the synthesis of saturnispol C (**54**) [71].

The synthesis of sorbicillins requires a dearomatization to afford a sensitive, cyclohexadienone diol. This challenging transformation has been implemented by Gulder and coworkers, using a SorbC monooxygenase enzyme, in order to afford sorbicillinoids which could then be elaborated to natural products including Saturnispol C (**54**), D, and Trichosorbicillin A (**Scheme 16**) [71]. Interestingly enough, the only requisite reaction to afford these three natural products was a Diels-Alder reaction, which was facile using the electron rich cyclic diene afforded by the dearomative hydroxylation of the enzyme under atmospheric conditions. One limitation of this report is potential scalability; reactions were below 0.15 mmol scale, though it is not clear whether this due to cost or a true limitation.

**Scheme 17:**
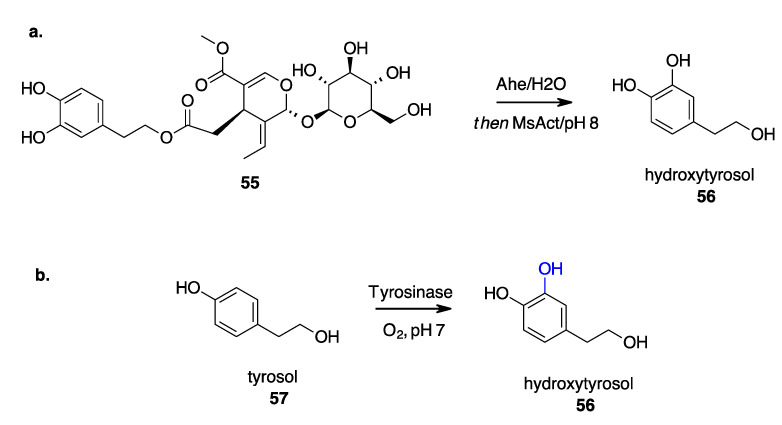
(**a**) Two-step chemoenzymatic hydrolysis in the synthesis of high demand antioxidant hydroxytyrosol (**56**). (**b**) One-step chemoenzymatic oxidation to yield hydroxytyrosol (**56**). [75,76].

The conversion of abundant natural compounds to other high-value natural products is a valuable path towards synthesizing them. Hydroxytyrosol (**56**) is a sought-after antioxidant with a high scale of demand and a deceptively simple chemical structure. Recently several patents and papers have been published for the synthesis of this compound among others, many of which are chemoenzymatic syntheses [72,73,74]. One such report by Pinto et al. leverages 10–20% of the mass of dry olive leaves isolated as intermediate 55 to form hydroxytyrosol (**56**), a potentially useful antioxidant compound (**Scheme 17a**) [75]. This is performed by sequential enzymatic hydrolysis of a hemiacetal moiety and an ester moiety using a glucosidase and an acyl transferase acting as an esterase.

As an alternative strategy, Pinto et al. published a constant-flow chemoenzymatic synthesis of hydroxytyrosol. Their method was to oxidize tyrosol (**57**) aerobically in the presence of a tyrosinase from *Agaricus bisporus*, in an ascorbic acid/phosphate buffer (**Scheme 17b**) [76]. Although unable to obtain complete conversions, they were able to design a facile flow-based separation method to afford pure hydroxytyrosol. The authors also demonstrated a flow-based chemoenzymatic acylation of tyrosol and hydroxytyrosol using sacrificial ethyl acetate, catalyzed by an immobilized acyl transferase MsAcT. A current limitation of this is scale: the maximum 0.25 mL/min flow rates were limited the yields obtainable in a 24 h period. This marriage of two frontier tactics in organic synthesis, flow and chemoenzymatic synthesis, is impressive. It is also an elegant solution to one of the classic issues of chemoenzymatic syntheses: low concentrations are common, which means it is difficult to make large amounts of material. Automated flow syntheses mostly sidestep this issue as the product is made without human involvement, and generally at a rate exceeding that of simply scaling batches.

## 3. Conclusions

Natural products continue to fascinate and inspire isolation, synthetic, and bioorganic chemists with their rich library of molecular complexity and biological applications. Pushing the boundaries of synthetic chemistry and biochemistry by using chemoenzymatic syntheses to create these molecules has become a field on to itself. As is the case in reaction methodology-based fields of synthetic chemistry, progress is achieved in incremental steps through the pioneering work of many scientists. Often the first efforts are accomplishments that have limitation in yield, scale, or substrate scope, but the ingenuity and persistence of researchers continues to advance the field. The discovery of new enzymes/reactions, improvement of yields and stereospecificity, and engineering of systems that utilize multiple enzymes, flow chemistry, and other emerging technologies is a testament to the talented scientists working in the field of chemoenzymatic synthesis of natural products. The molecular diversity and breadth of molecule classes to which chemoenzymatic synthesis is applied, as highlighted in this report, is truly remarkable and we look forward to the evolution and expansion of work in this area in the coming years.
